# Obituary

**Published:** 1867-10

**Authors:** 


### OBITUARY.

A SPECIAL meeting of the Pittsburg Dental Association was held at the office of Dr. J. S. King, Oct. 3d, 1867, to take some suitable action in regard to the death of Dr. HENRY BAKER; when a committee of seven was appointed to draft appropriate resolutions. The following resolutions were presented:

*Resolved*, That this Society show its affection for the virtues of our departed friend and brother, by placing on record these expressions of our bereavement and sorrow for his departed worth.

*Resolved*, That our sympathies are hereby tendered to the relatives and friends of the deceased in this sad and inscrutable dispensation of Providence.

*Resolved*, That a copy of these resolution be conveyed to the family of the deceased.

*Resolved*, That these proceedings be published in the Dental Journals.

All of which is respectfully submitted,

J. S. KING,

C. L. WUESTENBERG,

JAS. ORR,

G. W. SPENCER,

J. GREENAWALT,

M. S. KING,

M. E. GILLESPIE.

} *Committee.*

